# Microbiome-based therapies for Parkinson’s disease

**DOI:** 10.3389/fnut.2024.1496616

**Published:** 2024-11-06

**Authors:** Mudassir Alam, Kashif Abbas, Mohd Mustafa, Nazura Usmani, Safia Habib

**Affiliations:** ^1^Indian Biological Sciences and Research Institute (IBRI), Noida, India; ^2^Department of Zoology, Faculty of Life Sciences, Aligarh Muslim University, Aligarh, India; ^3^Department of Biochemistry, J.N. Medical College, Aligarh Muslim University, Aligarh, India

**Keywords:** gut microbiota, Parkinson’s disease, enteric nervous system, FMT, gut dysbiosis

## Abstract

The human gut microbiome dysbiosis plays an important role in the pathogenesis of Parkinson’s disease (PD). The bidirectional relationship between the enteric nervous system (ENS) and central nervous system (CNS) under the mediation of the gut-brain axis control the gastrointestinal functioning. This review article discusses key mechanisms by which modifications in the composition and function of the gut microbiota (GM) influence PD progression and motor control loss. Increased intestinal permeability, chronic inflammation, oxidative stress, α-synuclein aggregation, and neurotransmitter imbalances are some key factors that govern gastrointestinal pathology and PD progression. The bacterial taxa of the gut associated with PD development are discussed with emphasis on the enteric nervous system (ENS), as well as the impact of gut bacteria on dopamine production and levodopa metabolism. The pathophysiology and course of the disease are associated with several inflammatory markers, including TNF-α, IL-1β, and IL-6. Emerging therapeutic strategies targeting the gut microbiome include probiotics, prebiotics, synbiotics, postbiotics, and fecal microbiota transplantation (FMT). The article explored how dietary changes may affect the gut microbiota (GM) and the ways that can affect Parkinson’s disease (PD), with a focus on nutrition-based, Mediterranean, and ketogenic diets. This comprehensive review synthesizes current evidence on the role of the gut microbiome in PD pathogenesis and explores its potential as a therapeutic target. Understanding these complex interactions may assist in the development of novel diagnostic tools and treatment options for this neurodegenerative disorder.

## 1 Introduction

Parkinson’s disease (PD) is a progressive neurodegenerative condition defined by the degeneration of dopaminergic neurons in the substantia nigra pars compacta (SNc) that causes a variety of motor and non-motor symptoms (1). PD is becoming a severe public health concern with a global prevalence of roughly 6.1 million people in 2016 and a projected to be twice by 2040 (2). PD shows complex etiology, which includes both hereditary and environmental factors that contribute in the disease pathogenesis (3). Study reported that mutations in genes such as SNCA, LRRK2, PARK7, PINK1, and PRKN have been linked to familial forms of PD, whereas genome-wide association studies revealed over 90 risk loci for idiopathic PD (4). The human gut microbiome (HGM), which contains around 10^14^ bacteria, play crucial role in sustaining host health and affecting physiological systems such as the central nervous system (CNS). Gut-brain axis (GBA) is a bidirectional communication network between the gastrointestinal tract (GIT) and CNS, involving nervous, immune, and endocrine systems (5, 6). The gut microbiota (GM) present in the gastrointestinal tract (GIT) plays crucial role in maintaining the health of host by regulating cells in local as well distance organs, including brain (7). GBA facilitates bidirectional transmission that enables two-way communication between the gut and the host’s neurological system. Information is transmitted through brain networks, hormones and the immune system to facilitate the intestinal microbiota (8). According to epidemiological research, PD patients frequently experience gastrointestinal symptoms (GIS) such as constipation due to changes in the autonomic nervous system that slow down the movement of food through the digestive tract. (9). This finding is consistent with Braak’s theory, which postulates that PD disease may begin in the enteric nervous system (ENS) and then travel down the vagus nerve to the CNS (10). Molecular and cellular research has shown that Gut dysbiosis (GD) influences PD progression by various pathways, including GBA, Toll-like receptors (TLR), Humoral immunity response (HIR), α-synuclein accumulation and hypothalamic–pituitary–adrenal (HPA) axis (11). GD interferes with the local and systemic inflammatory states that result in compromised intestinal epithelial barrier integrity (IEBI). Simultaneously, GD causes disruptions in permeability of the brain parenchyma, which leads to neuroinflammation and neuronal cell malfunction (12). The progression of PD is increasingly linked to TLR-mediated immune responses, which result from the persistent activation of gut TLRs caused by microbial dysbiosis. There are studies indicating the involvement of TLR2 and TLR4 in PD pathogenesis (13). TLR2 and TLR4 are expressed by various cell populations throughout the gastrointestinal barrier, where they are activated in response to microbial byproducts and endogenous substances. Within the epithelial barrier, intestinal epithelial cells (IECs) and enteroendocrine cells (EECs) express TLR2 and TLR4, while in the submucosa, macrophages and dendritic cells do the same (14, 15). Smooth muscle cells in the muscular layer of the gut and throughout the enteric nervous system (including subepithelial and myenteric neurons and glia) also express these receptors. Apart from their role in innate immunity, TLR2 and TLR4 also regulate homeostasis and permeability of gut (16). The expression of TLR2 is influenced by gut microbes, and TLR2 is capable of detecting bacterial components such as lipoteichoic acid, lipoproteins, peptidoglycans, and bacterial amyloid (for example, curli protein) (17). Curli protein binds to and activates TLR2, leading to an increase in intracellular α-syn, which in turn triggers a neuroinflammatory response through the TLR2/MyD88/NF-κB pathway (18, 19). Similarly, the activation of TLR2 in the brains of PD patients leads to elevated levels of proinflammatory cytokines and microglial recruitment, intensifying neuroinflammation and α-syn expression. A strong correlation between these pathologies is indicated by the high TLR2 immunoreactivity observed in most α-syn-positive Lewy bodies (20, 21). Furthermore, a higher number of TLR4-expressing cells are observed in the colonic tissues of PD patients compared to healthy controls (22). HIR system is recognized to be one of the feasible pathways through which GD affects the brain, leading to the onset of PD (23). Highlighting the cooperative state of the microbiota and the innate mucosal immune system is crucial. The gut wall immune system comprises diverse immune cell populations such as IgA-producing plasma cells, γδT cells, and CD4+ T cells with a dominant Th1 or Th2 phenotype (24).

Recent research has indicated that the CD4+ T-cell population in the intestinal mucosa includes numerous Th-17 cells that generate Interleukin-17, as well as T-regulatory cells. Furthermore, it has been documented that there are IL-22-producing NK-22 cells present (25). α-synuclein is a protein that naturally occurs in healthy nerve cells and is primarily located in presynaptic terminals, where it helps regulate the function of synaptic vesicles and the release of neurotransmitters. Its molecular mass is around 14 kDa and consists of 140 amino acids (26, 27). Aggregation of α-synuclein and misfolding of normal cellular prion proteins (CPP), are important mechanism in PD pathogenesis (28). Accumulation of α-synuclein causes disruptions in cellular processes, which results in loss of dopamine-motor producing neurons and symptoms (29).

Changes in the makeup or function of gut bacteria could compromise the intestinal barrier, allowing prions to travel from the gut to the brain. Studies observed that gut bacteria produce metabolites such as lipopolysaccharides (LPS), and short-chain fatty acids (SCFAs) that are directly associated with the α-synuclein aggregation process (30). It has been demonstrated that LPS of Gram-negative bacteria can cause α-synuclein aggregation and set off inflammatory reactions (31). Gram-negative bacteria like *Escherichia coli, Pseudomonas aeruginosa, Klebsiella pneumonia,* and *Helicobacter pylori* stimulate TLR4, triggering immune responses that lead to the production of pro-inflammatory cytokines, chemokines, and oxidative factors. Increased blood endotoxins influence inflammatory cytokines that triggers the blood–brain barrier (BBB) and circumventricular organs (CVO), which in turn activate microglia, leading to loss of synapses and neurons. Moreover, Study conducted on mouse model reported that LPS can downregulate occluding (a protein in tight junction) in the intestinal epithelial cells and upregulate TNF-α, which promotes the expression of α-synuclein (32, 33).

Several research studies suggested that altered GM plays a crucial role in accelerating oxidative stress, inflammation and DNA damage. These factors may contribute to PD development (34). The comparison between germ-free and conventionally raised mice through whole-genome bisulfite sequencing revealed that the presence of commensal microbiota in the intestinal epithelium influences TET2/3-dependent DNA methylation changes at the regulatory elements of specific genes, which helps in the maintenance of gut balance. Thus, microbiota driven epigenetic reprogramming is crucial for preserving intestinal homeostasis (35). The acute gut inflammation caused by dextran sodium sulfate (DDS), exposure of the intestinal epithelium to microbiota results in abnormal DNA methylation and chromatin modifications at regulatory elements, similar to those seen in colitis (36). Study reported that early-life inflammatory stressors can increase gut permeability via downregulating E-cadherin expression (an epithelial junction protein), mediated by elevated expression of MicroRNA-155 (37). However, compounds possess antioxidant and anti-inflammatory properties such as cinnamon and turmeric have been found to shown protective effects by modulating E-cadherin-2 expression (38). A study observed that amino acids containing selenium such as selenocysteine and its derivative selenocystine, Alpha-methyl selenocysteine [(αMe)Sec] could improve DSS-triggered oxidative stress and intestinal inflammation in mice (39).

Increased intestinal permeability in PD patients has been linked to altered microbial populations, which cause bacterial metabolites and inflammatory mediators to translocate into the systemic circulation. This leaky gut characteristic might be part of the low-grade chronic inflammation (40). Research has demonstrated the importance of volatile SCFAs, specifically butyrate, in maintaining the integrity of the intestinal barrier. Due to a lack of SCFAs, α-synuclein can move from the stomach to the brain more readily, which heightened intestinal permeability and the pathological dissemination of the protein (41). Metagenomic studies demonstrated a clear distinction in GM makeup between PD patients and healthy controls. Difference between PD patients and healthy controls include a reduction in bacterial community that produce butyrate such as *Faecalibacterium prausnitzii*, and a rise in mucin-degrading species such as *Akkermansia muciniphila* (42). Rise of mucin degrading bacteria and decline of essential butyrate producing bacteria in gut environments might have profound effects on immunological response, host metabolism and the production of neurotransmitters (43). Dietary interventions such as nutritional supplements or specific diets are commonly used in clinical practices to restore essential gut flora and treat neurodegenerative disorders. For instance, a Mediterranean diet (Plant-based foods and essential fats) or a nutritious diet including fruits, vegetables, and fish seems to help maintain or decelerate cognitive decline (44).

Nevertheless, probiotics, prebiotics, and fecal microbiota transplantation (FMT) have been proposed microbiome-based treatments that have shown promise in preclinical models and early clinical studies (45). Studies demonstrated that the administration of certain probiotic strains reduces α-synuclein aggregation and motor impairments in the PD mouse model (46). Similar to this, Goya et al. (47) found that the probiotic strain PXN21 of *Bacillus subtilis* inhibits α-synuclein aggregation and clears aggregates in an established *Caenorhabditis elegans* model of synucleinopathy. Prebiotics has been shown to improve the immune function of the host, reduce gut inflammation, strengthen the colon, and decrease allergic reactions. However, ingesting prebiotics does not directly impose these effects; rather they provide their benefits in an indirect manner. It has been observed that prebiotics improve the mucosal barrier by promoting the growth of probiotics, which in turn can increase epithelial defense mechanisms (48).

The FMT has demonstrated remarkable efficiency in restoring the healthy GM and other diseases caused by GM perturbation, particularly *Clostridium difficile* infection. Some studies have found that FMT is safe and can improve motor and non-motor symptoms of PD (49, 50). An initial uncontrolled study with 6 PD patients, administering donor FMT through colonoscopy was found to be safe and led to enhanced motor and non-motor symptoms of PD after 6 months. However, long-term investigations with extensive randomized controlled trials are needed to evaluate the effectiveness and overall safety (50). A clinical pilot study suggests that FMT in mouse model of PD reduced GM alterations and decreased inflammation by activating microglia and astrocytes in the brain region of substantia nigra (SN) (51). Another study of PD mouse model induced with 1-methyl-4-phenyl-1,2,3,6-tetrahydropyridine (MPTP) revealed that FMT is capable of reducing expression of alpha-synuclein, inhibiting microglia activation and blocking TLR4/P13K/AKT/NF-KB signaling in the SN (52). These pathways significant role in the neuroinflammatory processes associated with PD. Inhibition of these pathways through interventions like FMT may offer therapeutic benefits in managing PD symptoms and slowing its progression.

This review article aims to deliver an extensive overview of the state of knowledge on the involvement of GM in PD pathogenesis and the developing avenue of microbiome-based therapeutics. Moreover, it examines the molecular pathways that trigger GBA in PD patients, assess the safety and effectiveness of several microbiome-targeted therapies, and address the obstacles and potential paths forward in this domain. Furthermore, this review also emphasizes on the potential of microbiome-based therapies for transforming the neurodegenerative pathways and therapeutic approaches in PD treatment.

## 2 Effect of the gut microbiome on neural interactions

**Figure 1:**
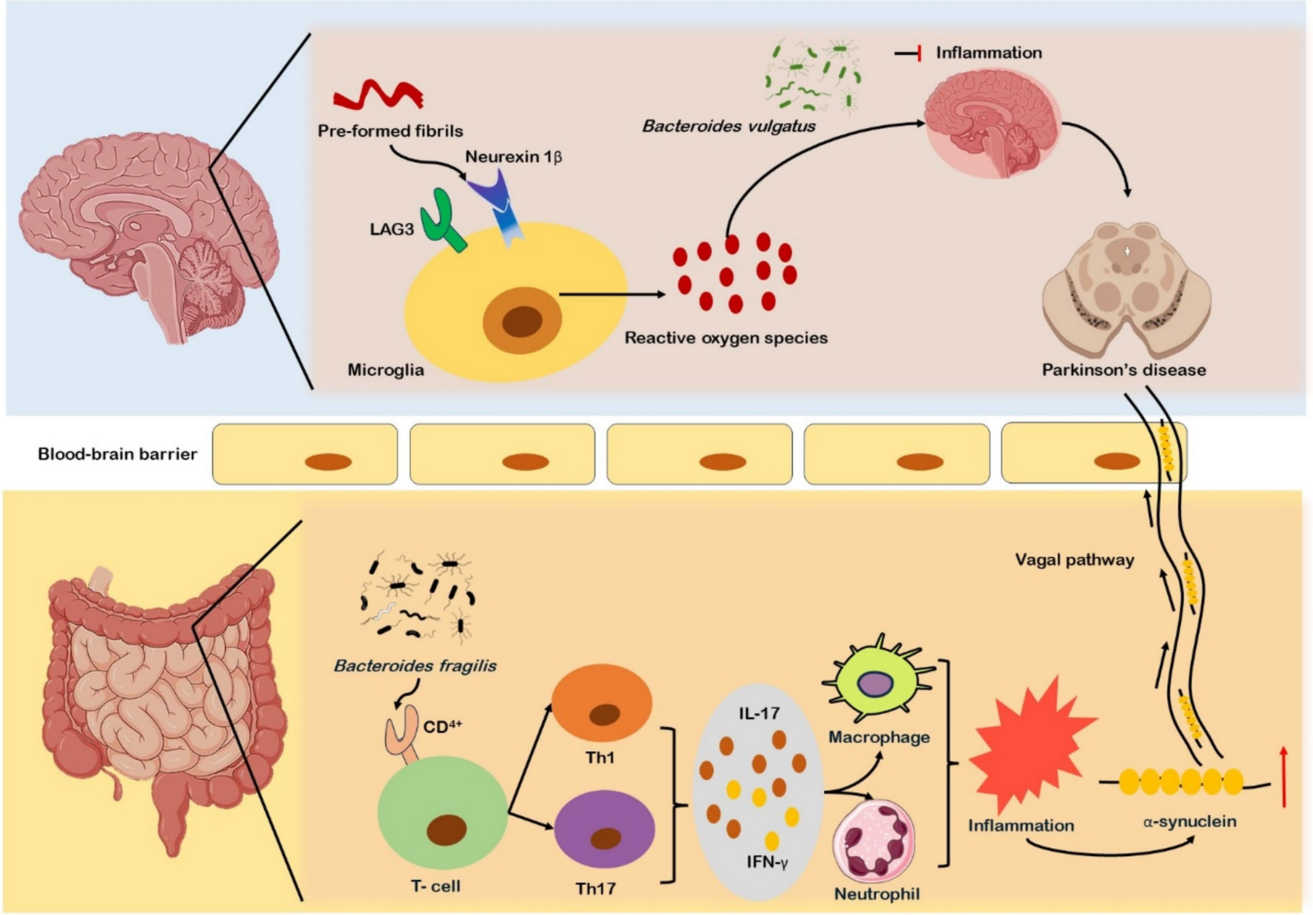
Gut microbiota alterations and their role in Parkinson’s disease (PD) progression. In PD, α-synuclein accumulates in enteric neurons before motor symptoms, with altered gut microbiota (GM) increasing oxidative stress and promoting α-synuclein aggregation. This aggregation may migrate to the brain via vagal and systemic pathways, leading to intestinal and systemic inflammation, blood–brain barrier (BBB) dysfunction, and microglial activation. Microbial species such as *Bacteroides vulgatus*, *Parabacteroides distasonis*, and *Lactobacillus salivarius* influence neuroinflammatory signaling. GM-induced CD4+ T cell activation triggers Th1/Th17 differentiation, cytokine release, and inflammation, promoting α-synuclein aggregation and its transference to the CNS. Elevated pro-inflammatory markers (TNF-α, IL-5, IFN-*γ*) are found in PD colonic biopsies.

The majority of the microbial species accommodated in the human body are found in the large intestines, where they can disrupt the availability and absorption of nutrients and may impact the homeostasis of the host system (53). The ENS communicates with the autonomic nervous system (ANS) and CNS through sensory and motor neurons as well as neurotransmitters. The vagus nerve’s afferent and efferent fibers are the primary source of the neurological circuitry that mediates this connection (54). Neurotransmitters such as dopamine, acetylcholine, serotonin, and some neuroactive chemicals such as short-chain fatty acids (SCFAs), can communicate with the brain via endocrine system (55). Colon biopsies performed on PD patients showed accumulation of α-synuclein in the colon’s enteric neurons before diagnosis of motor symptoms, which suggests α-synuclein may aggregate in the ENS before its dissemination to the brain (56). Moreover, as previous studies suggested that alterations in GM elevate oxidative stress, promoting α-synuclein aggregation within the intestines. The aggregated α-synuclein migrates to the brain through vagal or systemic pathways. Increased intestinal permeability could lead to systemic inflammation and BBB disruption, which may activate microglia in the brain (57). The binding and internalization of pre-formed fibrils (PFFs) to receptors, neurexin1β or Lymphocyte Activation Gene 3 (LAG3) activate microglia (Figure 1). Microglial cells show increased expression of LAG3, their activation leads to increased oxidative stress, resulting in neuroinflammation. This neuroinflammation facilitates further aggregation and propagation of α-synuclein contributes to neurodegeneration and the progression of PD (58). Microbial peptides and metabolic products are important for microglia function in the CNS, disturbance in microbiota producing essential metabolic products poses severe impact on inflammatory balance, which influences the progression and development of PD (59, 60). Microorganisms such as *Bacteroides vulgatus*, *Parabacteroides distasonis*, *Lactobacillus sali*var*ius*, and various *Clostridium* species, have been reported to alter the neuroinflammatory signaling that impacts the functioning of the brain (61). Research indicated that GM has a role in instigation of inflammation through activation of CD4+ T cell responses (62). On activation CD4+ T cells differentiate into Th1 and Th17 subtypes, releasing cytokines like IL-17 and IFN-*γ*, these cytokines further stimulate neutrophils and macrophages, resulting in the onset of intestinal inflammation (63). The inflammation initiated by Th1 and Th17 cells further promotes α-synuclein aggregation within ENS and its eventual transference to CNS (64) (Figure 1). Some findings from colonic biopsies of PD patients showed increased pro-inflammatory markers such as TNF, IL-5, and IFN-*γ* (65). The research demonstrated that CD4+ T cells are responsible for neurodegeneration, as CD4+ T lymphocytes have been found as key mediators in dopaminergic neuronal death in the MPTP mouse model of PD (66). In contrast, the absence of CD4+ T cells in immunodeficient mice resulted in reduced neuronal death (67).

## 3 Association between Parkinson’s disease and gut microbiota

The individuals with early-stage PD were found to have structures of α-synuclein in their colons that suggest the disease might originate in the gut first (68). The gastrointestinal issues in PD patients often begin before the onset of PD, which indicates a connection between intestinal disorders and the neurological diseases (69). *Helicobacter pylori* is a common bacterium that colonizes gastric mucosa and causes severe gastrointestinal issues in the gut (70). Studies have shown a significant correlation between the presence of *H. pylori* and the worsening of motor symptoms in PD patients (71). Research has indicated that the quantity of fecal tyrosine decarboxylase (TyrDC) is positively correlated with levodopa dose, whereas there is an inverse association between plasma levodopa levels and the presence of bacterial TyrDC genes in PD patients’ jejunum (72). *Enterococcus faecalis* is able to decarboxylate both levodopa and tyrosine; therefore, PD patients with high levels of *Enterococcus faecalis* experience decreased serum concentrations of levodopa (73). The presence of *Enterococcus faecalis* and its TyrDC-coding genes can therefore predict individual differences in levodopa metabolism within the complex gut microbiome (74). Above mentioned studies make *Enterococcus faecalis* is a key factor in levodopa bioavailability, thus targeting this bacterium could be a potential strategy to enhance the effectiveness of levodopa in treating PD.

Sulfate-reducing bacteria belonging to the genus *Desulfovibrio* (DSV) are anaerobic microorganisms which generate energy via reducing sulfate and producing a significant amount of sulfide (75). DSV is chiefly found in the human gut and is associated with inflammatory bowel disease (IBD) (76). Research using case control studies has confirmed that people with PD had higher relative abundances of *Desulfovibrionaceae* bacteria in their gut. DSVs are strongly associated with the onset and progression of PD (77). DSVs actively generate hydrogen sulfide (H2S) through sulfate reduction by utilizing sulfate as an electron acceptor during the respiration and increase H2S production, which causes severe damage to the CNS (78). H2S releases mitochondrial cytochrome c into the cytoplasm, which induces the production of α-synuclein free radicals and its polymerization (79). Furthermore, H2S is also associated with disruption of iron metabolism by increasing cytoplasmic iron level, which is critical for CNS homeostasis (80). Higher amounts of DSV have been found in the stool samples of PD patients, almost every stool sample from PD patients had positive results for the DSV-specific (FeFe)-hydrogenase gene (81). The quantity of DSV bacteria in fecal samples correlates with the severity of PD; hence, variations in DSV levels may serve as a preliminary marker of the advancement of PD.

## 4 Inflammatory processes and their impact on Parkinson’s disease

**Figure 2:**
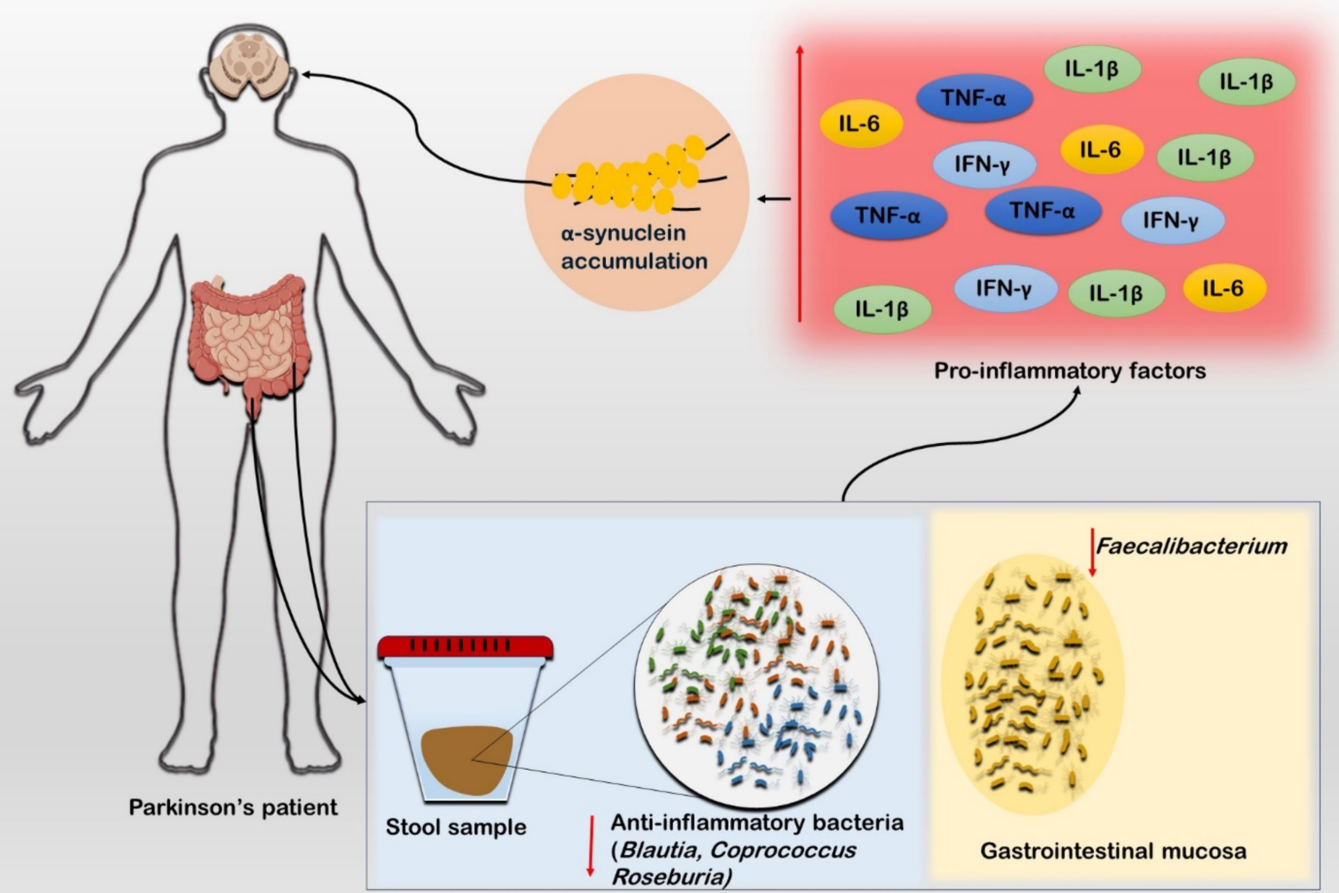
Role of inflammatory cascades in Parkinson’s disease. The stool sample from the PD patient showed diminished population of anti-inflammatory microbiota such as *Blautia*, *Coprococcus,* and *Roseburia*. Furthermore, the sample from gastrointestinal mucosa also exhibited low population of anti-inflammatory bacteria such as *Faecalibacterium*. Studies suggest that these both conditions are linked to higher concentration of the inflammatory markers such as TNF-α, IL-5, and IFN-γ in PD patients. The inflammatory markers further triggers α-synuclein production which exacerbates the ailing condition.

The aging-related inflammatory conditions are known as Inflammaging, which are significantly influenced by the GM alterations (82). Intestinal inflammation and PD are closely related, as evidenced by the elevated expression of inflammatory cytokines in the intestinal tissues of PD patients (83). Higher levels of pro-inflammatory cytokines and glial cell markers were observed, and stool samples had higher concentrations of inflammatory mediators such as TNF-α, IL-1β, IL-6, and IFN-*γ* in PD patients’ colon biopsy tissues, which indicate gastrointestinal inflammation (84) (Figure 2). It has been found that there is a change of anti-inflammatory bacteria in PD patients. A study revealed substantial reduction in *Blautia*, *Coprococcus*, and *Roseburia* genera in stool samples of PD patients (85). The systemic sub-inflammatory state of PD is induced by the relative abundances of *Verrucomicrobia* and *Bacteroides* which are linked to higher plasma levels of TNF-α and IFN-γ (86) (Figure 2). Young Pink1 knockout mice experience severe dyskinesia and striatal dopaminergic axon loss when exposed to Gram-negative bacteria that cause mild intestinal symptoms in adulthood. It suggests a strong interaction between intestinal microbes and intestinal inflammation in addition to a genetic predisposition to PD (87).

## 5 Toll-like receptor signaling in Parkinson’s disease

**Figure 3:**
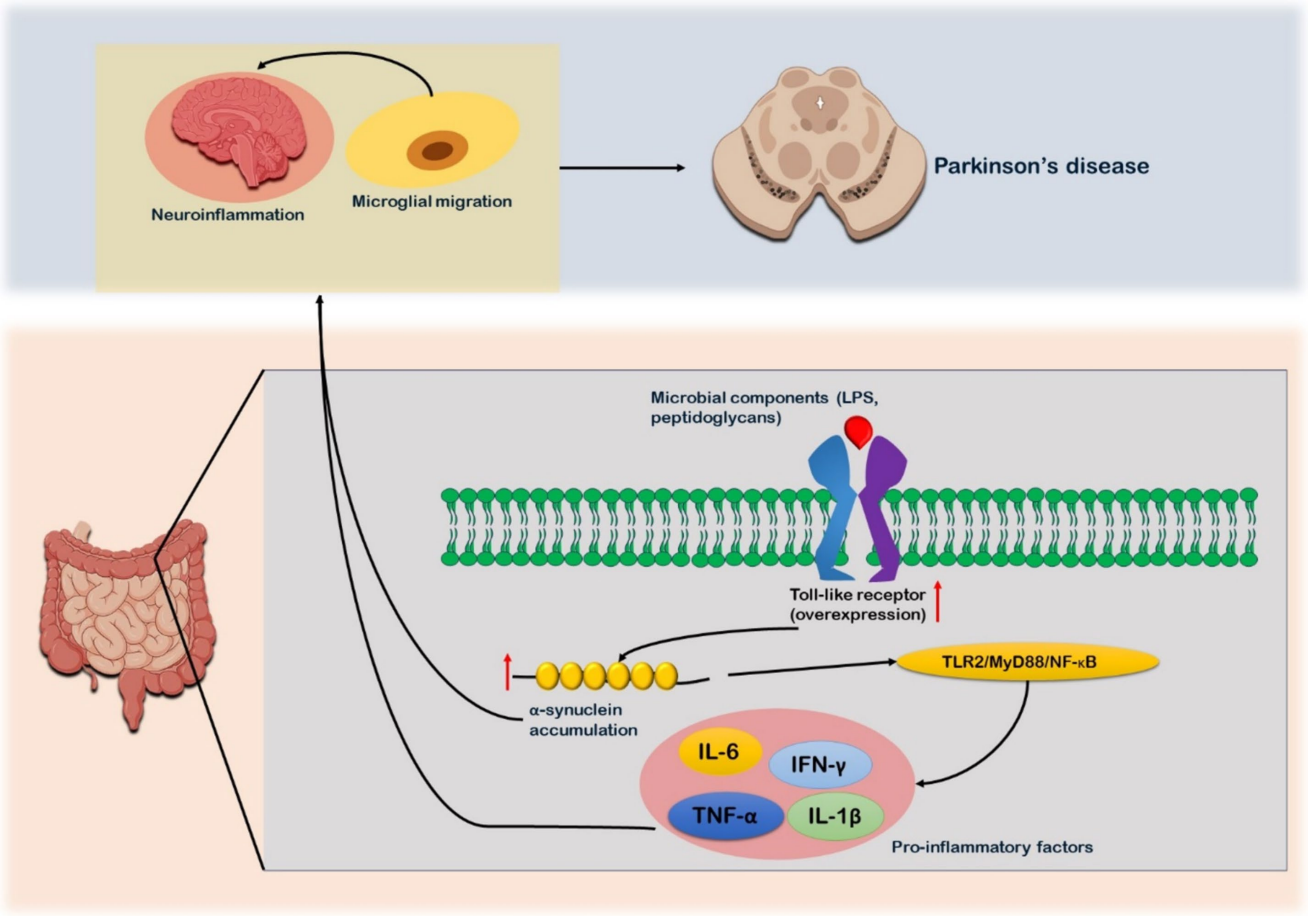
Toll-like receptor (TLR) signaling in relation to Parkinson’s disease. Microbial components such as lipoproteins, peptidoglycans, and lipoteichoic acid, which serves as ligand for the receptor. After their binding, TLR receptors are overexpressed as observed in PD patients. The binding triggers formation of α-synuclein. Production of α-synuclein triggers TLR2/MyD88/NF-κB signaling cascade which in turn leads to expression on pro-inflammatory factors such as TNF-α, IL-5, and IFN-γ. These pro-inflammatory factors further aggregate the α-synuclein accumulation. The α-synuclein and pro-inflammatory factors via enteric nervous system reaches the brain and cause neuro-inflammation and microglia migration. Microbial migration is also one of the contributing factors to the neuroinflammation.

Toll-like receptors (TLRs) are transmembrane pattern recognition receptor proteins that both preserve intestinal homeostasis and trigger the innate immune response by identifying invading microbial and viral components (88). A strong correlation between TLRs and PD has been shown in several investigations. TLR2 and TLR4 are overexpressed in the blood and brain tissues of patients with PD (89). TLR2, which identifies a variety of bacterial products, including lipoteichoic acid, lipoproteins, peptidoglycans, and bacterial amyloids like curli protein, can be expressed in response to the GM (13) (Figure 3). The binding to TLR2 raises intracellular α-synuclein levels, which in turn activates the TLR2/MyD88/NF-κB signaling cascade that causes a neuroinflammatory response (18). The activation of TLR2 in the brains of PD patients, results in elevated levels of pro-inflammatory cytokines and the migration of microglia along with intensification of neuroinflammation combined with elevated production of α-synuclein (20) (Figure 3). The majority of α-synuclein Lewy bodies possess high TLR2 immunoreactivity, suggesting a robust relationship between these pathologies (21). The colonic tissues of PD patients have shown to have more TLR4-expressing cells than those of healthy controls. The TLR4 signaling pathway is actively involved in the inflammation observed in the brains and intestines of PD patients. It is capable of recognizing endogenous chemicals and LPS from Gram-negative bacteria (90). TLR4 plays a crucial role in the removal of α-synuclein and triggers the microglial reactions (91). The effects of rotenone on intestinal barrier integrity, colonic α-synuclein levels, GFAP expression in the myenteric plexus, microglial activation in the nigrostriatal pathway, loss of dopaminergic neurons, and motor dysfunction are greatly attenuated in the presence of TLR4 (92).

## 6 Parkinson’s disease and its association with inflammatory bowel disease (IBD)

Several systematic reviews and studies mentioned that the risk of acquiring PD is greater in people with inflammatory bowel disease (IBD) (93). In comparison to healthy control, patients suffering with IBD show a reduced abundance of *Firmicutes* and a greater prevalence of *Enterobacteriaceae* (94). Several genetic risk factors that are common to both PD and IBD have been found via research into their association. PD and IBD are linked to mutations in the leucine-rich repeat kinase 2 (LRRK2) gene, which is implicated in microbial immunological signaling (95). There is strong evidence that LRRK2 is involved in immune cells and inflammatory illnesses (96). Research on animals suggests that Lrrk2 p.G2019S mice had more severe colitis than controls, which results in decreased motor function and death of dopaminergic neurons (97). LRRK2 mutations greatly worsen the inflammation in the brain and colon further influencing immunological responses and neuronal survival. Even during the prodromal period, when LRRK2 expression in the colon is noticeably raised, increased expression of LRRK2 has been observed in colon biopsies from PD patients, with expression levels corresponding with disease severity (98). The NOD2 gene has been repeatedly linked to PD susceptibility in studies that have linked it to Crohn’s disease (CD) closely (93). A study indicated that intronic single nucleotide polymorphism (SNPs) of NOD2 has been linked to PD risk (99). Moreover, the CARD15 gene SNP, linked to CD, is overexpressed in PD patients (100). Similar underlying processes are indicated by other known risk loci for both PD and IBD, which include lysosomal dysfunction (e.g., GALC and GPR65) and immunological response and microbial induction (e.g., the HLA locus) (101). Therapeutically, research has demonstrated that there is a correlation between IBD and PD. Individuals with IBD who were treated with anti-TNF biologics as part of their chronic anti-inflammatory regimen had a 78% lower risk of developing PD than those who did not get this medication (102). This indicates that PD may be prevented in part by suppressing peripheral inflammation.

## 7 Gut microbiota-based therapies for Parkinson’s disease

### 7.1 Probiotics, prebiotics, synbiotics, and postbiotics in PD management

Probiotics are live microorganisms that provide health benefits to the host when administered in sufficient amounts. Probiotics have gained much attention in the context of neurodegenerative diseases such as PD (103). The logic for their use in PD stems from the growing evidence that GD and altered GBA communication contribute to the disease pathogenesis. Probiotics possess strains of *Lactobacillus* and *Bifidobacterium* which are potential microorganisms that can restore microbial balance in the gut, ultimately reducing inflammation and improving GI functions, which are often impaired in PD patients (104). A study demonstrated that PD patients receiving a multi-strain probiotic supplement exhibited improvements in constipation and reduced levels of pro-inflammatory markers in the gut (105). Another study found that probiotic supplementation can modulate GM composition and potentially slow down the disease progression by reducing systemic inflammation (106). Probiotics also show beneficial effects through the production of short-chain fatty acids (SCFAs), these short-chain fatty acids possess anti-inflammatory properties and play a role in maintaining intestinal barrier integrity (107). Despite all of the beneficial data, the efficacy and potential of probiotics remain an area of research. Ongoing studies aim to refine the selection of bacterial strains, treatment duration, and dose requirements in different scenarios to maximize clinical benefits.

Prebiotics are non-digestible fibers that stimulate the growth and activity of beneficial gut bacteria. Prebiotics have been utilized as potential therapeutic agents in combating neurodegenerative disorders like PD (108). Prebiotics such as inulin, galactooligosaccharides (GOS), fructooligosaccharides (FOS) are known for promoting the growth and numbers of beneficial bacteria such as *Bifidobacteria* and *Lactobacilli* (109). These bacteria play vital roles in sustaining gut health and reducing inflammation in the gut. Studies suggested that prebiotics can influence the GBA and offer great neuroprotection in PD patients (110). A study demonstrated that prebiotics mimic probiotics as they also enhance the production of short-chain fatty acids (SCFAs) and possess anti-inflammatory properties that can strengthen the intestinal barrier function (111). By strengthening the gut integrity, prebiotics hinder the translocation of pro-inflammatory molecules and α-synuclein aggregates from the gut to the brain, which is a process in PD progression (112). Moreover, prebiotics administration in PD patients helps in restoration of gut microbial diversity, which often reduced due to dysbiosis in GM (113). Prebiotics treatment with sufficient doses has shown promising results in reducing gastrointestinal discomfort and systemic inflammation in PD patients (114). Synbiotics is a combination of both probiotics and prebiotics which is used as an advanced therapeutic approach for GBA dysfunction and other gastrointestinal disorders (115). Unlike probiotics or prebiotics, the synbiotics enhance the survival and activity of beneficial bacteria synergistically, thereby improving colonizing and growth of beneficial microbes in gut (116). Synbiotics have also been utilized to stimulate microbiota growth while increasing neuroprotective metabolites production such as SCFAs and butyrate (117). Research has shown that synbiotics administration in PD significantly reduces oxidative stress and neuroinflammation by influencing metabolic pathways of the gut microbiome (118). Another study highlighted that synbiotics may modulate the neuroinflammatory markers such as TNF-α and IL-6 that are found to be elevated in the case of PD (119). Proper calculated dosage of synbiotics offers a protective effect against neuronal damage. Unlike probiotic and prebiotic supplement administrations, the use of synbiotics potentially supports long-term gut microbial stability and provides sustained benefits for both gastrointestinal and central nervous system health (120). Postbiotics are the bioactive compounds that are produced by the fermentation of probiotics, it has attained great attention for their potent therapeutic role in several diseases, including neurodegenerative disorders such as PD (121). They consist of microbial metabolites such as short-chain fatty acids (SCFAs), lipopolysaccharides, enzymes, and peptides (122). It has been reported that SCFAs, chiefly butyrate, possess a neuroprotective effect, they promote anti-inflammatory pathways and enhance mitochondrial function in neuronal cells (123). These non-viable constituents employ beneficial effects on the host as they actively modulate immune responses by that inflammation is reduced, supporting the gut barrier integrity (124). The research highlights the importance of available interventions such as probiotics, prebiotics, synbiotics, and postbiotics. Several studies indicated the great promise of these components in alleviating the microbial dysbiosis that is implicated in PD patients. These microbiome-targeted interventions improve gut microbial balance, reduce inflammation, and support neuroprotection with no side effects (125, 126).

### 7.2 Fecal microbiota transplantation as a treatment for Parkinson’s disease

Fecal microbiota transplantation (FMT) is one of the comprehensive methods for GM restoration that involves the transfer of healthy GM from donors into PD patients (127). This practice has historical roots, a Chinese physician Ge Hong, employed this method to treat acute diarrhea and gastroenteritis (128). The FMT method is gaining significant attention for PD patients, although its clinical application is not yet very popular and accepted. There have been limited data and case studies to show strong outcomes (49). Some research suggested that the FMT method can reduce gastrointestinal consequences and improve both motor and non-motor symptoms in PD (129). A case study from China showed significant improvements in constipation and tremors associated with PD when FMT is followed (130). Another study also found that FMT administration through colonoscopy improved motor and non-motor symptoms after 6 months (129). A study of 2,010 patients for more than 3 months observed that FMT is a safe and effective therapy for treating gastrointestinal diseases (131). Moreover, this FMT method has shown great promise in improving sleep quality, anxiety, and depression in people suffering from PD (132). Studies on animals suggest that the FTM method can also be beneficial for PD patients, as this alters the microbial composition and modulates the immune response, which suppresses pathogen toxicity (133). In specified research it has been shown that FMT from the healthy mouse donor rectified GD in PD mouse model by increasing beneficial bacteria such as *Firmicutes* and *Clostridiales* (134). Moreover, the FMT therapy in PD mouse model enhanced physical performance and increased striatal serotonin and dopamine levels (135).

### 7.3 Dietary modification for management of Parkinson’s disease

Dietary understanding and its adjustments are an important approach for the management of PD. As the disease progression is linked to gastrointestinal dysfunction, inflammation, and GM alterations, several nutrition-based interventions play an essential role in improving both motor and non-motor symptoms associated with PD (136). Current research suggests that dietary modification with quality nutrition intake can reduce inflammation and enhance the quality of life (137). The Mediterranean and ketogenic diets, along with nutrients such as omega-3 fatty acids, vitamin D, and dietary fiber offer potential therapeutic benefits for PD progression and improving patient’s quality of life (138). The Mediterranean diet is the most recommended diet in management of PD, as this contains a high content of antioxidants, polyphenols, and omega-3 fatty acids (139). The Mediterranean diet, rich in vegetables, fruits, olive oil, whole grains, and fish, has been extensively studied for its neuroprotective effects (140). Mediterranean diet intake helps slow down the PD progression by reducing inflammation and promoting gut health by increasing microbial diversity (141). A thorough research analysis revealed that the Western diet exacerbated PD progression due to the poor quality of fiber, whereas the Mediterranean diet, high in fiber and polyphenols alleviated the symptoms of PD (142). A nutritious diet, like Mediterranean diet, has been demonstrated to increase beneficial gut flora, which is most required for maintaining gut health. Also, the Mediterranean diet can be used by gut flora to make essential metabolites such as SCFAs, these metabolites improve ENS function, thereby enhancing gastrointestinal motility (143). The ketogenic diet is very popular and has shown great promise in PD management. As the ketogenic diet contains moderate amounts of protein with high fat and low carbohydrate (144). Ketogenic diet helps reduce oxidative stress and improve mitochondrial function, which are compromised in PD patients (145). The ketogenic diet increases ketone bodies such as beta-hydroxybutyrate, which provides an alternate energy source to neurons, thus offering neuroprotection and reduced neuroinflammation (146). Clinical trial data have shown that the ketogenic diet can improve both motor and non-motor symptoms in PD patients and enhance the quality of life (147). Along with Mediterranean and ketogenic diet, several nutrients have also been investigated thoroughly for their potential role in PD. Increased intake of Omega-3 fatty acids has been evident in reducing neuroinflammation and slower neurodegeneration in PD patients (148). A study found that PD patients given high dietary Omega-3 fatty acids exhibited less oxidative stress, reduced production of α-synuclein, and improved cognitive function (149). Moreover, supplementation of Omega-3 fatty acids has been an excellent therapeutic approach to reintroducing beneficial gut flora (150). Similarly, the vitamin D effect was investigated in PD management, and found that vitamin D supplementation improved gut health, behavior, and cognition in PD patients (151). Caffeine is a natural stimulant found in coffee, tea, and various other foods and has been studied for its neuroprotective role, especially in PD (152). Epidemiological and experimental studies suggest caffeine helps improve motor symptoms and reduce the risk of PD progression (153). A related study highlighted that caffeine inhibits adenosine A2A receptors, found in the striatum (a region in the brain that controls movement), which enhances dopaminergic neurons to maintain the level of levodopa (154). Caffeine protects neurons from oxidative damage via increasing antioxidant enzymes, which reduce the production of reactive oxygen species (ROS) (155). Moreover, caffeine has been shown to inhibit chronic inflammation, its anti-inflammatory nature downregulates the pro-inflammatory cytokines and microglial activation, thereby protecting neuronal damage (156). Dietary fiber intake regulates gastrointestinal dysfunction, which is a prevalent issue in PD patients. Adequate fiber intake is of utmost benefit for gut environment and tackling gastrointestinal problems. Fiber-rich foods modulate GM and induce SCFA production to enhance anti-inflammatory properties (157).

## 8 Assessment of clinical trials, challenges, and future directions

Clinical trials investigations on microbiome based therapies for PD have shown promising outcomes. However, there is limited data and few clinical trials are there to support MBT in PD patients. One remarkable study directed at the Army Medical University (AMU) in China carried out a randomized, placebo-controlled trial involving 56 participants suffering from mild to moderate symptoms of PD [Hoehn and Yahr (H&Y) stages 1–3]. H&Y scale classifies severity of PD associated motor symptoms (158). The clinical trial ran from February to December 2019. Participants were splits into two groups. First group received FMT while second group were given a placebo. The results suggested that FMT group improved PD related autonomic symptoms, which were measured by the Movement Disorder Society Unified Parkinson’s Disease Rating Scale (MDS-UPDRS). This trial also reported remarkable reduction in total scores at the end, which was (*B* = −6.56, *p* < 0.05). Moreover, FMT was found to improve GI disorders and a surge in microbial diversity within the gut (130). Thereby, FMT administration could enhance the effectiveness of existing PD treatments with no adverse effects.

Another clinical trial was conducted at Ghent University Hospital between December (159) and December 2022, known as GUT-PARFECT trial. This double-blind placebo-controlled study included participants of 50–65 years age, and was experiencing early-stage PD (H&Y stage 2). Participants were randomly divided to give nasojejunal FMT from either healthy donors or their own stool. After 12 months results showed a significant decline in MDS-UPDRS score and improvement in both motor and non-motor symptoms in participants. This completed clinical trial is registered and can be found on ClinicalTrials.gov with (NCT03808389) (49). Moreover, a randomized double-blind placebo-controlled trial was conducted to evaluate the effects of probiotics on movement and metabolic factors associated with progression of PD. Total 60 participants were enrolled in the study, that were equally divided into two groups (*n* = 30 each group). First group received a daily dose of 8 × 10^9 CFU of probiotics, while other received a placebo for a period of 12 weeks. The MDS-UPDRS scores were recorded before and after the intervention. The results indicated that probiotic consumption led to a significant decrease in MDS-UPDRS scores compared to the placebo, that was recorded (−4.8 ± 12.5 vs. +3.8 ± 13.0, *p* = 0.01). Additionally, probiotic supplementation was associated with reductions in high-sensitivity C-reactive protein (−1.6 ± 2.5 vs. +0.1 ± 0.3 mg/L, *p* < 0.001). This trial suggests that a 12-week course of probiotic supplementation resulted in beneficial effects on both movement and other selected metabolic factors in individuals with PD (160). Thus, probiotics therapy could serve as a useful adjunct treatment for addressing the PD symptoms and associated metabolic disturbances. The clinical trial can be accessed on (ClinicalTrials.gov Identifier: IRCT2017082434497N4).

A recent clinical randomized trial study by Filip et al. assessed the safety and efficacy of colonic single-dose anaerobically prepared FMT in 45 PD patients with a follow-up period of 12 months. They concluded that FMT was found to be safe, and did not result in significant improvements in PD symptoms compared to the placebo. Their placebo group showed significant increase in dopaminergic medication and clinical improvements (161). The outcome of this trial suggests additional investigations into the effects of bowel cleansing and donor microbiota composition to establish strong results. This clinical trial can be found at clinicalTrials.gov Identifier: NCT04854291. A small study utilizing M-SHIME^®^ (a simulator of the human intestinal microbial ecosystem) demonstrated that probiotic supplementation could significantly alter bacterial composition and improve gastrointestinal health in PD patients. Ghyselinck et al. (162) used M-SHIME^®^ to determine the efficacy of probiotic supplementation in restoring bacterial composition in PD patients. They concluded that PD influenced by GD can be treated with supplementation of properly formulated probiotics as a useful adjunct. Since their study was limited by small number of subjects (6 subjects: 3 PD and 3 control) and shorter duration (48 h), the robust results could not be inferred (162).

The clinical implications of these findings are substantial, and suggest that targeting GM through FMT and other microbiome-modulating therapies like prebiotics and probiotics may not only alleviate GI symptoms associated with PD but also enhance SCFAs, and anti-inflammatory cytokines (IL-6, IL-10) production, while decreasing pro-inflammatory cytokines and chemokines (MCP-1 and IL-8) (48). The increased level of anti-inflammatory factors greatly tackles with inflammation, and strengthens the gut barrier integrity leading to improve quality of life (QOL) (163). It is well acknowledged that microbiome based therapies have shown remarkable results in PD management. However, further large-scale clinical trial studies are necessary to strengthen and establish standardized treatment protocols for microbiome-based therapies in PD.

There are numerous obstacles linked to microbiome-driven treatments in PD. Microbiome therapeutics show promise in terms of success but often encounter several challenges. Identifying the right microbes to target the complexities of diseases is the primary obstacle in microbiome therapeutics (164). Various therapeutic approaches require different microbial strains, depending on their ability to thrive in the body. The colon and caecum are effectively colonized by *Bacteroides* sp. *Lactobacillus* sp. and *E. coli* Nissle are successful in populating the small intestine. *Lactobacillus lactis* lacks the ability to colonize the intestine (165). Therefore, the suitability of the probiotic used for treatment is determined by the disease biogeography. Before selecting them for treatment, it is essential to thoroughly characterize microbes based on their functional benefits. Additionally, microbiome therapeutic research studies were primarily carried out using rodent models and *C. elegans* model, which shows variability in various aspects (166). Thus efforts are required for large human trials. The stability and robustness of the clinically relevant microbial strains ensure successful microbiome therapeutics. Regulatory challenges for microbiome therapies are of great concern. There is a need for clear guidelines on safety and efficacy assessments on microbiome therapies. Use of FMT raises ethical questions regarding donor selection and the potential transmission of pathogens or other unintended consequences from donor microbiota (167). Thus establishing ethical frameworks for such therapies is crucial as they move toward clinical application.

Advancement of biotechnology and microbiomic therapies has encouraged the use of modulatory therapies in clinical settings. Although research efforts have established the efficacy of microbiome therapeutics, still more research is required to fully grasp the microbiome and how it interacts with the host in order to advance the concept of microbiome therapeutics into clinical trials and develop a roadmap for effective treatment. The use of bacterial suspensions may impose a risk to patients, through entry of pathogens within the recipient; thus, strategies to avoid contamination and risk need to be developed. Moreover, prior introducing bacteria to patients, it is necessary to conduct a thorough genomic analysis of the bacterial populations in order to distinguish disease-specific signature microbes from those found in healthy individuals. Efforts should be made to create pills containing only one type of microbe to enhance the immune response and improve patient treatment. Therefore, it is necessary for microbiome therapeutic companies to collaborate with pharmaceutical industries in order to enhance the effectiveness of the treatments. The findings from the clinical trials on gut bacteria should be further investigated for autoimmunity and neurological disorders in order to advance the field of microbiome therapeutics.

## 9 Discussion

The complex and multidimensional role of gut flora play important role in the onset and progression of PD. GM imbalance or GD are strongly correlated with the progression and development of neurodegenerative disorders, specifically PD. Emerging research highlighted that GM alterations such as declined beneficial bacteria communities or increased harmful gut mucin-degrading species can initiate pathogenesis of several diseases, including PD (168). It has been well established that GM can affect brain trough GBA, which is a bidirectional communication between the gut and the brain, involving the immune, endocrine and nervous system. GM can affect brain through GBA in several ways that include neurotransmitter production, immune system modulation, ENS disruption and intestinal barrier modulation. Generally GM breaks down host’s food to fulfill its nutritional requirements, along with they generate metabolites like neurotransmitters or their precursors, which impact existed neurotransmitters level in brain. For instance, increase of *Akkermansia, Catabacter, Lactobacillus, Bifidobacterium, Bifidobacteriaceae, Ruminococcaceae, Verrucomicrobiaceae*, and reduction in *Roseburia, Faecalibacterium, Prevotellaceae, Blautia, Coprococcus*, and *Lachnospira* have been noted in individuals with PD (159, 169).

The presence of *Enterococcus faecalis* in PD patients is linked to the metabolism of l-DOPA and dopamine (170). In a PD mouse model study, it was found that giving berberine orally increases tyrosine hydroxylase activity in *Enterococcus faecalis*, leading to higher production of l-DOPA, a dopamine precursor. As a result, l-DOPA crosses the blood–brain barrier and gets converted into dopamine in the brain, which could reduce the symptoms associated with PD (171).

The increased levels of inflammatory cytokine and chemokine genes observed in the intestinal tissues of PD patients suggest a strong connection between PD and intestinal inflammation (85, 90). Furthermore, elevated levels of markers for glial cells (GFAP and Sox-10) and multiple pro-inflammatory cytokines are found in colon biopsy samples from individuals suffering from PD (65). Likewise, higher levels of different inflammatory substances (such as IL-1β, IL-6, IFN-γ, and TNF-α) found in the fecal samples of individuals with PD suggest the existence of inflammation in the gastrointestinal tract (84, 172). These inflammatory alterations enhance the host’s vulnerability to immune dysfunction and autoimmune reactions (173).

It is a widely recognized fact that PD affects both the CNS and the ENS. Studies on individuals with PD and animal subjects have revealed damage to neurons and glial cells in the ENS (174). Multiple research teams have documented the presence of Lewy-like abnormalities in biopsied enteric neurons of individuals with PD. Degeneration of neurons in the myenteric plexus and submucosal plexus with α-synuclein deposits has been documented in individuals with PD (175). Several mechanisms have been put forwarded to explain GD-assisted PD development; one such mechanism includes increased intestinal permeability or the leaky gut, which is very prevalent in the case of PD.

When gut barrier is compromised, it allows some bacterial products and inflammatory mediators into the bloodstream that directly or indirectly influences inflammatory cascades in brain. Bacterial elements such as LPS and amyloid protein curli have been shown to enhance α-synuclein aggregation (176). Kelly et al. (177) found that administering low doses of LPS led to a gradual rise in α-syn expression, resulting in intestinal permeability, primarily in the large intestine of mice. *E. coli* and other Gram-negative bacteria are capable of producing curli fibers (178).

The molecular mimicry of PD may result from an extracellular amyloid generated by gut bacteria. For instance, curli, an amyloid protein possessing property of human disease-related amyloids is secreted by *Escherichia coli* through biosynthetic processes (179). Curli may trigger the innate immune system and facilitate α-syn accumulation, which exacerbate neuroinflammation (180).

Many therapeutic approaches that target GM have shown great promise in managing and treating PD. The clinical use of Probiotics, prebiotics, synbiotics, and postbiotics offers a potential solution for restoring gut microbial balance and improving gut health. Some beneficial bacteria such as *Lactobacillus* and *Bifidobacterium* are of great importance, as they are crucial in keeping the gut environment healthy by reducing oxidative stress and inflammation. Tsao et al. (181) showed that in a PD-like model, *Lactobacillus salivarius* AP-32 reduced oxidative stress and inflammation, raised serum antioxidant activity, and enhanced SCFAs levels in fecal samples. Wang et al. (182) observed that *L. plantarum* DP189, is able to slow down the neurodegenerative process induced by α-synuclein in the SN of PD mice by reducing oxidative stress, inhibiting proinflammatory reactions, and restoring GM. Marsova et al. (183) discovered that *Lactobacillus* is able to decrease the amount of oxidative stress (reactive oxygen species) in the PD nematode model by controlling the Nrf2/ARE pathway. The evolutionary basis underlies Nrf2’s role in controlling antioxidant defense, it remains highly conserved in all vertebrates. Mouse Nrf2 shows 83.4% DNA homology and 82.5% protein homology when compared to human Nrf2 (184).

The FMT is another well-documented method for restoring the gut ecosystem, despite its positive outcome; further extensive research is needed to determine its long-term effects on PD patients. The potential role of Mediterranean, ketogenic, and nutrition-based diets in PD has been well documented in various research studies. The very high inter-variability in GM composition creates challenges in the development of standardized microbiome-based interventions to achieve optimal efficacy in this case an individual’s unique microbial profile is necessary. Additionally, the interaction between microbiome-targeted therapies and traditional PD treatments require careful evaluation to ensure safety and maximize benefits. Research should focus more on identifying specific microbial signatures or metabolites that can serve as early biomarkers for PD detection. Long-term and large-scale clinical trials are essential to establish the efficacy of microbiome-based interventions in different stages of PD. As gut microbiome represents a fundamental frontier in PD experiments and research, while many scientific points remain unclear. A huge body of evidence supports the gut-brain axis, GD directly or indirectly impacts gastrointestinal environment, which impacts the neuronal functioning in the brain. Further comprehensive research focusing on untouched areas of GBA may provide a better management strategy for PD and significantly improve patients’ quality of life.
